# Implementation of the Toronto guidelines for childhood cancer staging: experience from 65 population-based cancer registries participating in the BENCHISTA project

**DOI:** 10.1016/j.esmorw.2026.101185

**Published:** 2026-11-09

**Authors:** A. Lopez-Cortes, D. Burns, L. Botta, F. Didonè, A. Cañete Nieto, E. Desandes, L.L. Hjalgrim, Z. Jakab, C.A. Stiller, G. Gatta, K. Pritchard-Jones, BENCHISTA Project Working Group, Peter Baade, Leisa O'Neil, Florencia Moreno, Agustina Chaplin, Monika Hackl, Ruth Ladenstein, Elizabeth Van Eycken, Nancy Van Damme, Carlos A. Lima, Walmiro Ramos, Lucrecia Aline Cabral Formigosa, Luciana Ferreira dos Santos, Gil Patrus Mundim Pena, Juliana Nativio, Cyntia Asturian Laporte, Cristiana Santos de Menezes Miranda, Cristiane Bastos Daniel, Raimunda Nonata de Paulo, Donaldo B. Veneziano, Angela Pontes de Aquino, Paulo Cesar Fernandes de Souza, Yana Lopes, Rejane de Souza Reis, Zdravka Valerianova, Dobrin Konstantinov, Sumit Gupta, Jason D. Pole, Alexandra Airhart, Oscar Ramirez, Elvia Grillo, Jan Stary, Jaroslav Sterba, Jeanette Falck Winther, Keiu Paapsi, Brigitte Lacour, Emmanuel Desandes, Jacqueline Clavel, Claire Poulalhon, Meike Ressing, Claudia Trübenbach, Eleni T. Petridou, Evdoxia Bouka, Zsuzsanna Jakab, Miklós Garami, Rocco Galasso, Giuseppe Sampietro, Patrizia Piga, Marcella Sessa, Milena M. Maule, Carlotta Sacerdote, Paola Ballotari, Marilena Ceresola, Ettore Bidoli, Antonina Torrisi, Rosalia Ragusa, Enrica Santelli, Ilaria Cozzi, Luca Boni, Magda Rognoni, Rosalba Amodio, Federica Manzoni, Francesco Cuccaro, Danila Bruno, Antonio G. Russo, Federico Gervasi, Maria L. Gambino, Pietro Seghini, Maria L. Michiara, Luciana Mangone, Claudia Cirilli, Stefano Ferretti, Federica Zamagni, Fabio Falcini, Eugenia Spata, Sonia Manasse, Paolo Coccia, Fabrizio Stracci, Daniela Piras, Pasquala Pinna, Francesca Bella, Andreas Klaus Bulatko, Adele Caldarella, Teresa Intrieri, Tiziana Scuderi, William Mantovani, Maria Adalgisa Gentilini, Manuel Zorzi, Stefano Guzzinati, Tracey Brennan, Tomohiro Matsuda, Kayo Nakata, Kyu-Won Jung, Marika Gurupta, Jelena Rascon, Miriam J. Azzopardi, Tom Borge Johannesen, Aina H. Dahlen, Einar Stensvold, Joanna Didkowska, Ursula Wojciechowska, Anna Raciborska, Ana M. Ferreira, Gabriela Caldas, Maria José Bento, Mihaela Bucurenci, Daniela Coza, Miroslava Makohusova, Gabriela Valachova, Vesna Zadnik, Tina Žagar, Arantza Lopez de Munain, Fernando Almela-Vich, Noura Jeghalef-El Karoui, Aina Romaguera Palomé, Rafael Marcos Gragera, Maria José Sanchez, Nuria Aragones, David Parra-Blazquez, Maria Dolores Chirlaque, Ricardo J. Vaamonde, Marcela Guevara, Elena Pardo, Rafael Peris-Bonet, Adela Cañete Nieto, Marià Carulla, Päivi Lähteenmäki, Claudia E. Kuehni, Sophia Della Valle, Shelagh M. Redmond, Otto Visser, Henrike Karim-Kos, Sarah Stevens, Lucy Irvine, Anna Gavin, Deidre Fitzpatrick, Damien Bennett, David S. Morrison, Karen Smith, Claire Manson, Dyfed Wyn Huws, Stephanie Smits, Dawn Allan, Angela Polanco, Riccardo Capocaccia, Andrea Di Cataldo, Meric Klein, Maciej Trojanowski

**Affiliations:** 1 UCL GOS Institute of Child Health, UCL Developmental Biology & Cancer Research Department, London, UK; 2 Epidemiologia Valutativa e Sviluppo innovativo Traslazionale, Department of Translational Research, Epidemiology and Data Science, Fondazione IRCCS Istituto Nazionale dei tumori, Milan, Italy; 3 Paediatric Oncology and Hematology Unit, Department of Pediatrics, Obstetrics and Gynecology, Hospital UiP La Fe, Valencia, Spain; 4 Spanish Registry of Childhood Tumours (RETI-SEHOP), University of Valencia, Valencia, Spain; 5 National Registry of Childhood Cancers, CRESS, UMRS 1153, INSERM, Paris-Cité University, Paris, France; 6 National Registry of Childhood Solid Tumors, Institut de Cancérologie de Lorraine, Vandœuvre-lès-Nancy, France; 7 Department of Paediatrics and Adolescent Medicine, University of Copenhagen, Rigshospitalet, Copenhagen, Denmark; 8 National Childhood Cancer Registry (NCCR), Hungarian Pediatric Oncology Network (HuPON), Budapest, Hungary; 9 Department of Pediatrics, Semmelweis University, Budapest, Hungary; 10 National Disease Registration Service, Transformation Directorate, NHS England, London, UK; 11 Department of Statistics and Quantitative Methods, University of Milano-Bicocca, Milan, Italy

**Keywords:** barriers, childhood cancer, implementation, population-based cancer registry, routine practice, Toronto stage

## Abstract

**Background:**

Population-based cancer registries (PBCRs) are central to measuring childhood cancer outcomes and informing global health policy. The International Benchmarking of Childhood Cancer Survival by Stage (BENCHISTA) project stimulated participating PBCRs to assign stage at diagnosis according to the international Toronto Stage Guidelines (TGs). This study aims to provide a quantitative assessment of operational findings and structural barriers limiting routine TGs implementation within PBCRs participating in the first phase of BENCHISTA.

**Patients and methods:**

Seventy-three PBCRs participating in the first phase of BENCHISTA were invited to complete a semistructured survey assessing operational practices, data collection capacity, and structural and operational barriers to routine implementation of the TGs and associated non–stage-prognostic (NSP) variables across six solid childhood tumours. Sixty-five PBCRs from 23 countries provided valid and complete answers.

**Results:**

Before BENCHISTA, 85% PBCRs reported no routine collection of TGs. Following participation, 51% indicated that they would continue staging all childhood cancers. The principal barriers to TGs assignment were workload (38%), lack of linkage with clinical sources (32%), and time required for data retrieval (29%). Molecular NSP variables—particularly rhabdomyosarcoma fusion markers and medulloblastoma subgroups—were the most difficult to capture due to inconsistent pathology reporting and limited registry access to molecular data. Fewer than half had systematic collection of treatment and recurrence data.

**Conclusions:**

This multinational survey provides a quantitative assessment of barriers limiting routine TGs implementation in PBCRs. Sustaining consistent data collection will require targeted investment in workforce capacity, improved interoperability between clinical and registry systems, and standardisation of clinical, molecular, and pathology reporting frameworks.

## Highlights

• Increased Toronto Stage collection by cancer registries after joining BENCHISTA.
• Molecular biomarker capture remains an important access gap in population-based data.
• Resource constraints are the dominant barrier to staging and enhanced data capture.
• Treatment data collection is mostly high but structural barriers limit integration.
• Follow-up data infrastructure varies noticeably between registries.

## Introduction

Before 2014, no internationally agreed staging system suitable for use by population-based cancer registries (PBCRs) across all childhood cancer types existed. The TNM (tumour–node–metastasis) staging system, used for most adult cancers, is inappropriate for most paediatric malignancies. Although tumour-specific staging systems exist within clinical trial groups, these vary between diagnostic groups and provide no common framework for population-based surveillance of stage at diagnosis.^1^, ^2^

To address this, an international group of experts developed the Toronto Paediatric Cancer Stage Guidelines (TGs).^2^ The guidelines adopt a two-tiered approach enabling registries with different levels of data access and resources to participate. Subsequent updates extended the framework to include non–stage-prognostic (NSP) factors and molecular, histological, and cytogenetic variables required for risk stratification and interpretation of survival differences.^3^, ^4^, ^5^, ^6^

The International Benchmarking of Childhood Cancer Survival by Stage (BENCHISTA) project was established to stimulate the implementation of the TGs by paediatric and general PBCRs. It focused on six solid tumours: neuroblastoma, Wilms tumour, medulloblastoma, Ewing sarcoma, rhabdomyosarcoma, and osteosarcoma, diagnosed within a recent 4-year period (2014-2017) to maximise ease of data collection.^7^, ^8^, ^9^ At the end of BENCHISTA phase 1, a survey of participating registries explored the operational and structural barriers limiting routine collection of high-resolution data required to apply the TGs; its findings form the basis of this report.

## Materials and methods

PBCRs participating in BENCHISTA phase 1 (January 2021 to January 2024) were invited to complete a survey on data collection experiences and the project’s impact. The survey comprised 19 questions across three items: identification (four questions), experiences within BENCHISTA (eight questions), and current registry practices (seven questions) (Appendix S1, available at https://doi.org/10.1016/j.esmorw.2026.101185).

The survey was developed and administered in Qualtrics software (Qualtrics XM.) (University College London licensed, https://qualtrics.ucl.ac.uk/). After piloting among BENCHISTA Project Management Team members, the instrument was refined for readability and accessibility before distribution to PBCR representatives in the Project Working Group on 14 November 2024. Two e-mail reminders were sent, with a response deadline of 18 December 2024. To accommodate representatives who could not respond within this period, the survey was reopened from July to November 2025 to capture any outstanding responses. This extended window was agreed upon to maximise the response rate across our large international network.

Survey data were exported from Qualtrics and processed in Excel (Qualtrics XM). Responses to multiple-choice items were standardised and frequency analyses were carried out across all response options. Categories were developed iteratively on the basis of the most frequent response patterns. Response frequencies were calculated for all survey domains, including Toronto stage assignment, NSP collection, other optional clinical variables (i.e. treatment modalities, recurrence, and cause of death), follow-up data availability, and structural and operational barriers. Denominators were constant except for the NSP questions, which one registry did not answer.

Ethical approval for research use of data was provided by the institutional review boards of University College London and Fondazione IRCCS Istituto Nazionale dei Tumori di Milano, acting as joint data controllers. All procedures complied with relevant laws and local ethics committees when required.

## Results

Of 73 PBCRs from 27 countries participating in BENCHISTA phase 1, 65 PBCRs from 23 countries (Table S1, available at https://doi.org/10.1016/j.esmorw.2026.101185) completed the survey (89% response rate).

### Toronto stage: baseline practices and impact of BENCHISTA

Before BENCHISTA, the routine collection of Toronto stage was uncommon. Fifty-five registries (85%) reported no routine collection at diagnosis, eight (12%) reported routine collection, and two (3%) reported collection limited to selected paediatric tumours.

Following project participation, 33 registries (51%) indicated that they would continue to assign TGs for all childhood cancers, 11 (17%) indicated they would continue assigning TGs for selected paediatric tumour types only, 16 (25%) indicated that they would not continue assigning TGs, and five (8%) were uncertain (Figure 1).

**Figure 1:**
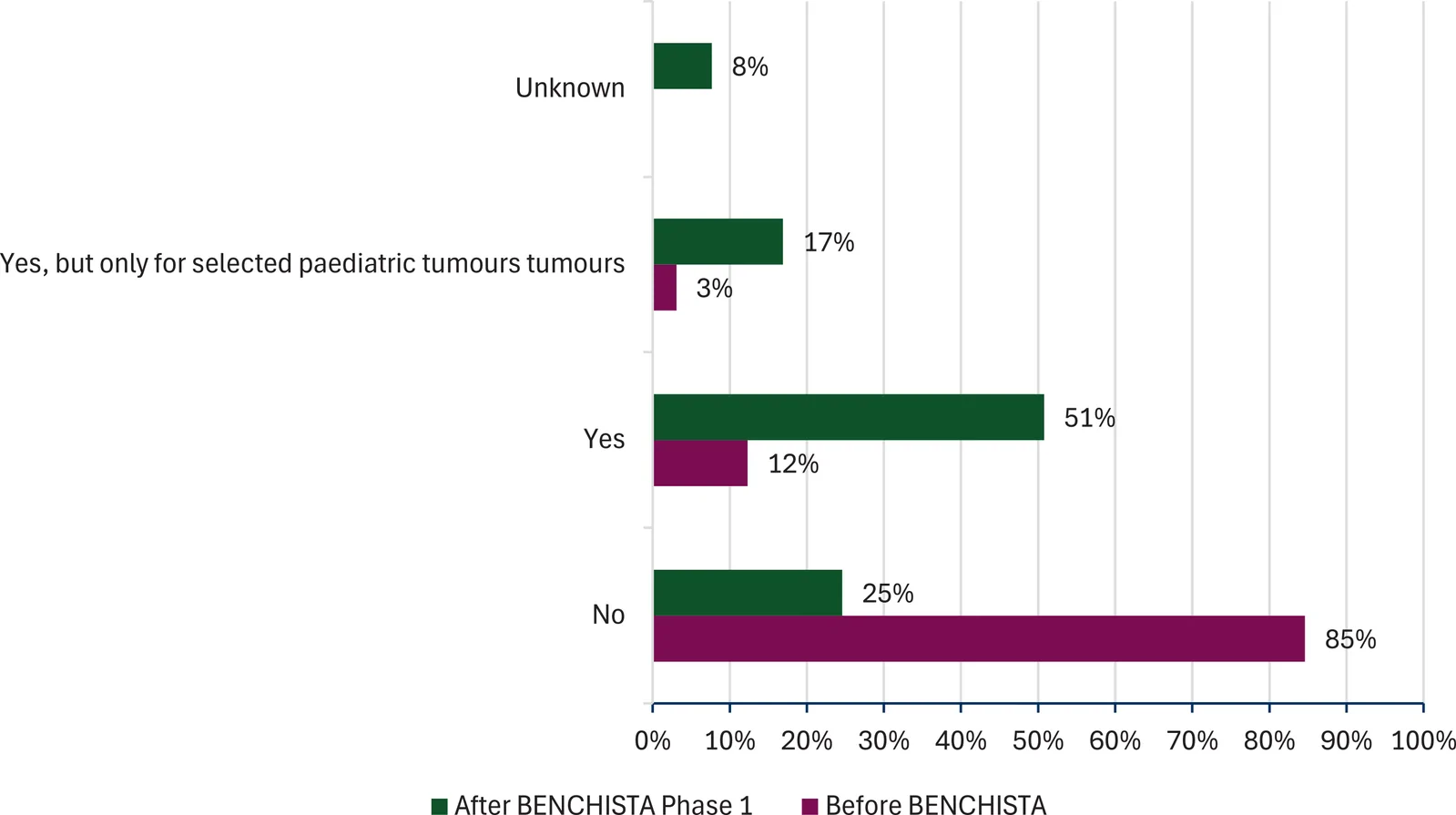
**Routine collection/assignment of Toronto stage at diagnosis before and after BENCHISTA.** Percentages may not sum to 100% because of rounding. BENCHISTA, International Benchmarking of Childhood Cancer Survival by Stage.

Thirty registries (46%) reported that BENCHISTA participation led to modifications in how they assign tumour stage at diagnosis, whereas 35 (54%) reported no change. Among those reporting changes, the most common were adoption or expansion of the TGs (22 of 30) and improved data collection quality and standardisation (11 of 30). Figure S1 (available at https://doi.org/10.1016/j.esmorw.2026.101185) summarises additional improvements.

Availability of clinical support to resolve ambiguous cases or data sourcing varied. Twenty-seven registries (42%) relied on a paediatric cancer specialist involved in the patient’s care, 23 (35%) relied on data managers and/or research assistants, and 14 (22%) relied on a clinician (usually a cancer specialist supporting PBCR work). Six registries reported no clinical support, and seven reported local or *ad hoc* input from clinicians (non-specialists) or PBCR experts.

### NSP and optional variables, current practice, and challenges to data collection

The BENCHISTA project requested additional variables labelled as ‘optional’. These included the tumour-specific NSP factors defined in the TGs for four of the six tumour types studied, together with variables on treatment modalities used, recurrence, and cause of death.

During phase 1, NSP collection was primarily based on active case-level retrieval. Forty-six registries (71%) collected NSP factors through specific clinical sources such as pathology or laboratory reports, clinical notes, or administrative databases; 33 (51%) used information already held within the registry; and six (9%) relied on linkage with clinical registries or trial databases.

Out of 64 respondents for this question (Appendix S1, page 3, question 7, available at https://doi.org/10.1016/j.esmorw.2026.101185), the NSP factors most frequently reported as challenging to capture were rhabdomyosarcoma fusion markers (FKR-PAX-3: 41, 64%; FKR-PAX-7: 40, 63%) and medulloblastoma subgroup markers (SHH: 37, 58%; WNT: 35, 55%). Neuroblastoma MYCN amplification (24, 38%), and Wilms tumour anaplasia (21, 33%) were less frequently reported as difficult to retrieve (Figure 2).

**Figure 2:**
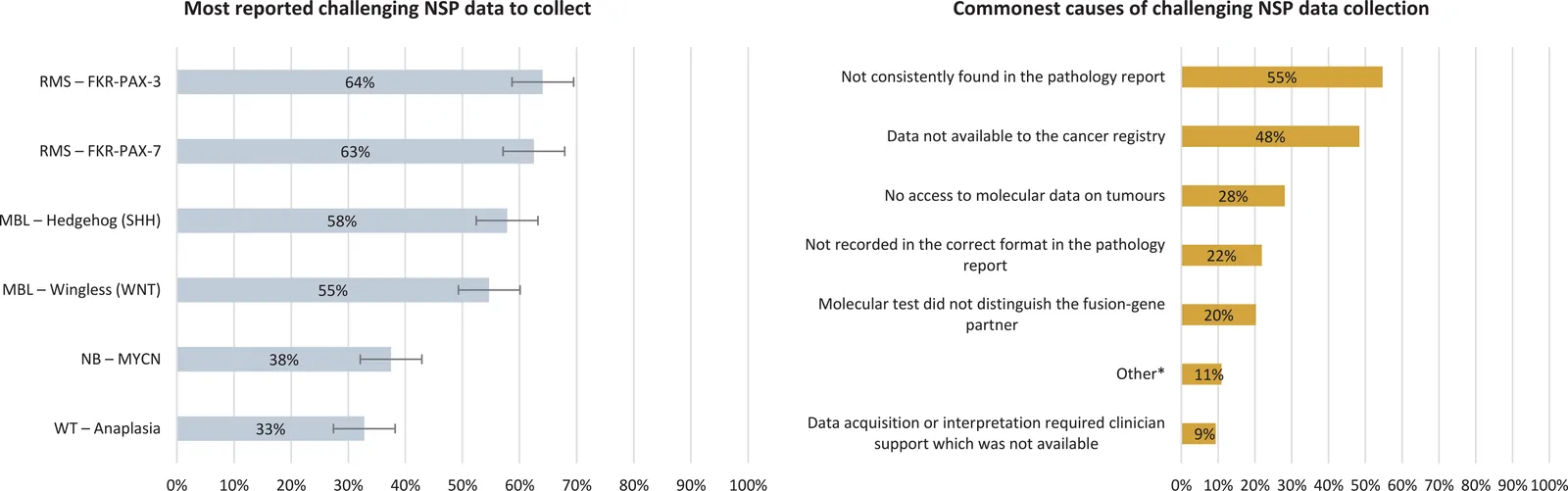
**The most challengingnon-stage prognostic (NSP)factors to collect in BENCHISTA andcommonestcauses reported.** One registry did not answer Q7 and Q8 (Appendix S1, page 5, available at https://doi.org/10.1016/j.esmorw.2026.101185), which are represented in the graphs. The total denominator used for these two items was 64. For Q7 and Q8, multiple answers were permitted, which is why percentages do not sum to 100%. The graphs show the most frequently mentioned responses when disaggregated. Bars included in the figure “Most reported challenging NSP data to collect” are standard error bars. BENCHISTA, International Benchmarking of Childhood Cancer Survival by Stage; MBL, medulloblastoma; NB, neuroblastoma; Q, question; RMS, rhabdomyosarcoma; WT, Wilms tumour. ∗Other causes included information not adequately recorded, molecular testing carried out in private laboratories requiring extra funding to access its results, and NSP factors not always performed.

Systematic collection of treatment modality data for all childhood cancers was reported by 30 registries (46%), with four (6%) collecting for selected tumours only. Thirty-one (48%) reported no systematic collection. Nineteen (29%) routinely collected recurrence or disease progression data for all childhood cancers, whereas 7 (11%) collected only for selected tumours. Fifty-three (82%) indicated feasibility of routinely collecting cause-of-death data.

Participants were asked about the feasibility of continuing to collect data for the optional variables after BENCHISTA phase 1. Fifty-five (85%) registries considered surgery feasible, 53 (82%) chemotherapy, and 52 (80%) radiotherapy. Feasibility was lower for immunotherapy (40, 62%) and recurrence (41, 63%) (Figure 3).

**Figure 3:**
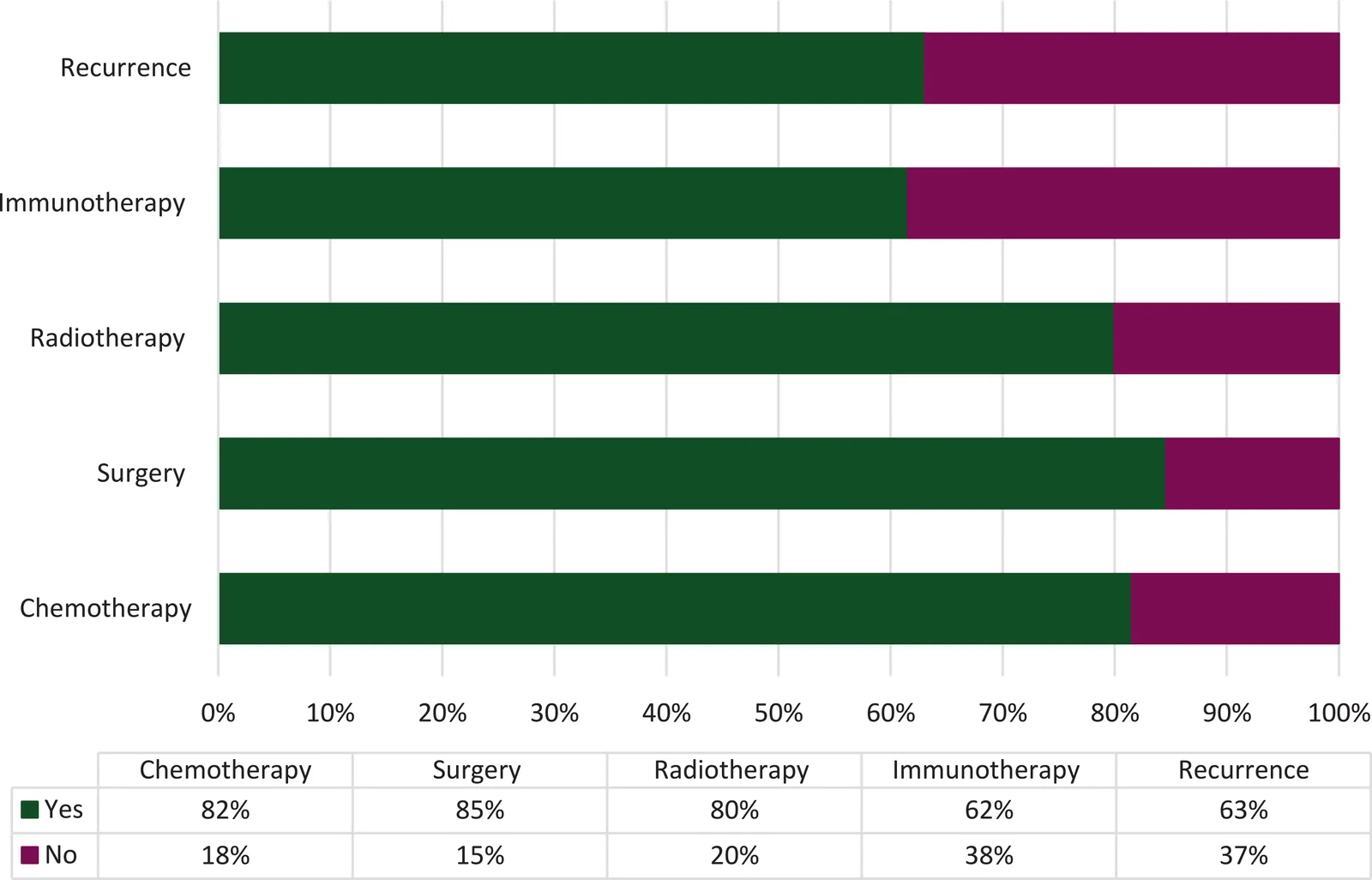
**Feasibility of sustained treatment and recurrence data collection in PBCRs following BENCHISTA phase1.** BENCHISTA, International Benchmarking of Childhood Cancer Survival by Stage; PBCRs, population-based cancer registries.

Forty registries (62%) reported that they routinely collect complete follow-up data; five (8%) do not routinely collect follow-up data but have access to it; five (8%) routinely collect follow-up data but it is not always complete; three (5%) reported neither routine collection nor access to follow-up data. Three (5%) had access but did not collect routinely and nine (14%) reported other parameters including data linkage with other sources to collect vital status.

Follow-up data is achieved through linkage with national mortality databases or death registries (43 of 65, 66%), hospital/clinical records (25 of 65, 38%), administrative databases (24 of 65, 37%), national or regional health databases (23 of 65, 35%), and electronic health records (18 of 65, 28%).

### Barriers to data capture during BENCHISTA phase 1

Barriers reported were primarily structural and operational. For the Toronto stage assignment, the most common were workload (25 of 65, 38%), lack of linkage with clinical sources (21 of 65, 32%), time required to complete the task (19 of 65, 29%), and lack of workforce (18 of 65, 28%). Limited training (15 of 65, 23%), topography/morphology coding challenges (11 of 65, 17%), and funding constraints (8 of 65, 12%) were reported less frequently (Table 1). We noted that workload, lack of workforce, time required to complete the task, and funding constraints might not be mutually exclusive and could reflect overlapping aspects of resource insufficiency. Taken together, 38 of 65 respondents (58%) cited at least one barrier within this cluster.

**Table 1:** Reported limitations/challenges in assigning Toronto stage to six solid tumours in BENCHISTA phase 1 and areas of improvement

Barrier (survey Q3)	*n* (%)	Suggested area of improvement
Workload	25 (38)	Increased workforce capacity
Other^a^	25 (38)	See Table S2, page 8 (available at https://doi.org/10.1016/j.esmorw.2026.101185), for a full breakdown of the ‘other’ category responses
No linkage with clinical sources	21 (32)	Standardise reporting formats^b^ Electronic health record linkage Enhanced interoperability
Time required to complete the task	19 (29)	Increased workforce capacity
Lack of workforce	18 (28)	Increased workforce capacity
Lack of or limited training	15 (23)	Continuously available training^c^/educational programme on Toronto stage
Specific challenges related to topography/morphology coding	11 (17)	Continuously available training^c^ Linkage with clinicians to assess complex cases and query resolution
Funding	8 (12)	Strong governance framework to support cancer registries

Multiple answers were allowed for this question, which is why percentages do not sum to 100%. The suggested areas for improvement are those discussed in the article and reflect the authors’ opinions; registries were not asked to propose solutions.

BENCHISTA, International Benchmarking of Childhood Cancer Survival by Stage; Q, question.

a The ‘other’ category (*n* = 25) primarily described difficulties in accessing clinical records and incomplete clinical data, topics that might be consistent with the predefined ‘no linkage with clinical sources’ barrier.

b Including the online CanStaging+ tool (https://canstaging.org/), which has specific guidance notes for applying Toronto staging to paediatric cancers.

c For example, the video training workshops focused on six solid childhood tumours included in the BENCHISTA Project, developed by members of the team and supported by lead expert clinicians with the purpose of supporting cancer professionals in achieving standardised and reliable implementation of the Toronto Guidelines.

The core barrier to NSP factors collection was a data reporting and access gap. Thirty-five of 64 responding registries (55%) indicated that NSP factors were not consistently found in pathology reports; 31 (48%) reported that data were not available to the registry; 18 (28%) had no access to molecular data; 14 (22%) reported that information was not correctly recorded or formatted in pathology reports; 13 (20%) registries indicated that molecular tests did not distinguish the specific fusion-gene partner; six (9%) indicated that data acquisition or interpretation required clinician support, which was not available; and seven (11%) mentioned other reasons, representing systemic issues beyond registry-level control (Figure 2).

## Discussion

This multinational survey provides the first quantified assessment of the barriers limiting routine collection of data required to apply the TGs to the six BENCHISTA solid tumours.

All registries successfully implemented the TGs at diagnosis within the BENCHISTA project, with reported feasibility of continuing to assign Toronto stages for all, or selected childhood solid tumours—33 (51%) and 11 (17%) respectively. However, sustained routine collection of these variables remains constrained by workforce capacity, access to clinical information systems, and governance frameworks.^10^, ^11^

The survey also examined current registry practices and feasibility of capturing NSP factors, as well as other optional variables required to interpret variation in treatment and survival. Although cause of death was routinely captured by most registries, the systematic collection of treatment and recurrence data was less common. These findings indicate that many PBCRs still rely on case-level abstraction from clinical documentation rather than automated data flows from health information systems.^12^

Operational constraints were frequently cited as barriers to assigning the TGs and extracting NSP factors. Assigning TGs at diagnosis often requires the reconstruction of diagnostic information from pathology reports, imaging, and clinical notes. This process is time-intensive and difficult to sustain without dedicated staff resources. Paediatric oncology specialists are often relied on to interpret complex diagnostic staging information or resolve ambiguous cases. Without automated interfaces between registry databases and clinical systems, manual data extraction increases workload and limits the routine incorporation of high-resolution variables into registry workflows.

Cancer registry access to clinical information varied, with many reporting limited or no linkage with clinical information systems, preventing direct transfer of pathology, imaging, or laboratory data. Molecular variables were frequently unavailable to registries or inconsistently reported. Rhabdomyosarcoma fusion status and medulloblastoma subgroup markers were among the variables most often cited as difficult to capture. These markers were only incorporated into the World Health Organisation paediatric tumour classification since 2016 (medulloblastoma) and later for rhabdomyosarcoma fusion genes.^13^, ^14^, ^15^, ^16^ Structured reporting of routine diagnostic variables in modern paediatric oncology, pathology, and laboratory systems is often lacking. Consequently, molecular data required for Toronto Guidelines may exist within clinical systems but remain inaccessible for population-based surveillance, prompting efforts to include key molecular markers in registry datasets and establish formal links with clinical sources.

Governance frameworks further influence how clinical information reaches registries. In some jurisdictions, legal or institutional constraints limit registry access to full clinical records or diagnostic databases.^17^, ^18^ Although the European General Data Protection Regulation was intended to facilitate data exchange for research purposes, its interpretation varies considerably across countries and institutions, creating diverse conditions for cross-border data sharing. Models of data collection among PBCRs also differ. For instance, the Belgian Cancer Registry relies on clinicians across specialist centres to assign Toronto staging directly and submit it to the registry, whereas registries such as the English National Cancer Registration and Analysis Service benefit from national health data feeds but may face operational constraints when reconstructing detailed staging information outside of project-funded activity. Such variation—reflecting diverse national legislative and institutional contexts—highlights the need for harmonised data access policies.

These findings suggest that PBCRs operate under diverse structural configurations that generate predictable challenges for data capture and no single intervention will enable routine implementation of Toronto staging across all settings. Registries with access to clinical information systems may benefit primarily from increased workforce capacity,^19^ whereas those lacking such access require improvements in interoperability and automated linkage with clinical systems.^17^ Without improvements in workforce capacity, data linkage, and governance frameworks, incorporating these optional variables into routine surveillance will remain challenging.^20^ Where governance frameworks restrict direct access to clinical records, clinician-assigned staging or structured data submission may represent pragmatic alternatives. This is outlined further in Table S2 (available at https://doi.org/10.1016/j.esmorw.2026.101185).

As a limitation, this study relied on self-reported information, reflecting perceived rather than measured operational practice. Data collection spanned a two-window time period and registry heterogeneity and variation in resources may limit generalisability. Future research should validate current findings against registry data and evaluate interventions designed to improve structured clinical data capture.

This multinational study provides the first overview of barriers limiting routine implementation of TGs in PBCRs. Although many registries successfully integrated Toronto staging within BENCHISTA,^8^, ^9^ sustained routine collection of stage and associated prognostic variables remains constrained by insufficient workforce capacity, limited linkage with clinical information systems, and governance restrictions on access to diagnostic data. These barriers similarly impede the systematic capture of treatment, recurrence, and molecular data required for risk stratification. Addressing these challenges across diverse registry contexts might require strengthened workforce capacity, improved clinical registry system interoperability, and structured high-resolution data reporting into population-based cancer surveillance—essential for positioning PBCR data as a cornerstone of health data information systems and for establishing a robust international benchmark for childhood cancer outcomes that can inform health policy at scale.

## Informed consent

We provided verbal information to members of the BENCHISTA Project Working Group about taking part in the survey; participation was voluntary and completing the survey was taken as consent. All the data included in the paper come from the survey results. The BENCHISTA team discussed the survey information and shared the manuscript and relevant materials with the Project Working Group. Further information has been discussed with core and Project Management Team members from BENCHISTA. The privacy rights of participants have been observed.

## Ethical approval

This study was conducted in compliance with the relevant laws, regulatory frameworks, and guidelines applicable to the research settings. This study was conducted in accordance with the ethical approval for research use of data under the BENCHISTA Project, granted by the institutional review boards of University College London and Fondazione IRCCS Istituto Nazionale dei Tumori di Milano, which acted as joint data controllers. All procedures complied with relevant laws and local ethics committee requirements. The BENCHISTA Project received approval from both University College London (UCL) and Fondazione IRCCS Istituto Nazionale dei Tumori di Milano (INT), with approval numbers 19963/001 (UCL) and 146–21, 27 May 2021 (INT).
