# Supplementary material for: Implementation of the Toronto guidelines for childhood cancer staging: experience from 65 population-based cancer registries participating in the BENCHISTA project
Source: ESMO Real World Data Digit Oncol. 2026 Nov 9;14:101185. doi: 10.1016/j.esmorw.2026.101185 (PMC13770634; doi:10.1016/j.esmorw.2026.101185)
Supplement: Supplementary file 1 [file mmc1.docx]

**Supplementary material**

[Appendix S1. Survey questions	2](#_Toc244250802)

[Table S1. Participating countries in BENCHISTA providing a valid entry to the survey	6](#_Toc244250803)

[Figure S1. Additional changes reported by PBCRs in assigning tumour stage at diagnosis after participation in BENCHISTA	7](#_Toc244250804)

[Table S2. Outline of challenges/limitations reported and potential areas of improvement	8](#_Toc244250805)

[Table S3. BENCHISTA Project Working Group	9](#_Toc244250806)

# Appendix S1. Survey questions

**Data collection experiences from current participating cancer registries and the impact of BENCHISTA**

Identification

*Q1. Country*

*Q2. Cancer Registry Name*

*Q3. Role of representative completing the survey*

*Q4. Email address*

Questions related to the BENCHISTA Project

*Q1. Before the BENCHISTA project, did your cancer registry routinely collect Toronto Stage at diagnosis?*

*[Single answer]*

□ Yes

□ Yes, but only for selected paediatric tumours (please specify which ones in the comment box)

□ No

*If YES, please specify from which year your cancer registry has been collecting routinely Toronto Stage at diagnosis*

□ Open field answer

*Q2. After BENCHISTA, would your cancer registry/centre continue to assign the Toronto stage to paediatric childhood cancers?*

*[Single answer]*

□ Yes, for all childhood cancers

□ Yes, but only for selected paediatric tumours (please specify which ones in the comment box)

□ No

□ Unknown

*Q3. Which limitations or challenges were encountered when retrieving incidence data and assigning Toronto Stage to the six solid tumours included in BENCHISTA Phase 1?*

*[Multiple choice answer]*

□ Funding

□ Workload

□ Lack of workforce

□ Lack or limited training

□ Time required to complete the task

□ Specific challenges related to topography/morphology coding

□ No linkage with clinical sources

□ Other (open field answer)

*Q4. Has participation in the BENCHISTA Project led to any modifications in how your registry assigns tumour stage at diagnosis?*

*[Single answer]*

□ Yes

□ No

*If YES, please specify the main changes you have experienced after your participation in the BENCHISTA project*

□ Open field answer

*Q5. Which sources did you use to collect the BENCHISTA non-stage prognostic factors (NSPs)?*

*[Multiple choice answer]*

□ From data sources already held within the CR

□ Through specific data collection from regular data sources available to the CR. e.g., clinical sources (pathology reports, laboratory reports, clinical notes) and/or administrative databases

□ Through linkage (direct or indirect) with clinical registries/trial databases, etc

□ Other (open field answer)

*Q6. If you require clinical support to collect data or confirm ambiguous cases, who is normally the professional supporting you in terms of query resolution or data sourcing?*

*[Multiple choice answer]*

□ Data manager/Research assistant

□ Clinician (usually paediatric cancer specialist involved in the patient’s care)

□ Clinician (usually paediatric cancer specialist who supports the work of the CR for all cases)

□ Other (open field answer)

*Q7. For the purpose of the BENCHISTA project, which was the most challenging NSP data to collect?*

*[Multiple choice answer]*

□ Neuroblastoma – MYCN

□ Wilms Tumour – Anaplasia

□ Medulloblastoma – Wingless (WNT)

□ Medulloblastoma – Hedgehog (SHH)

□ Rhabdomyosarcoma – FKR-PAX-3

□ Rhabdomyosarcoma – FKR-PAX-7

*Q8. What were the reasons why collecting these NSP was challenging or difficult:*

*[Multiple choice answer]*

□ Not consistently found in the pathology report

□ Not recorded in the correct format in the pathology report

□ Molecular test did not distinguish the fusion-gene partner

□ No access to molecular data on tumours

□ Data acquisition or interpretation required clinician support which was not available

□ Data not available to the cancer registry

□ Other (open field answer)

Questions related to the cancer registry’s current practice

*Q1. Regarding optional variables, does your registry collect data on treatment in a systematic way?*

*[Single answer]*

□ Yes, for all childhood cancers

□ Yes, but only for selected paediatric tumours (please specify which ones in the comment box)

□ No

*If YES, please specify from which year your cancer registry has been systematically collecting data on treatment*

□ Open field answer

*Q2. Does your registry routinely collect recurrence/progression of the disease*

*[Single answer]*

□ Yes, for all childhood cancers

□ Yes, but only for selected paediatric tumours (please specify which ones in the comment box)

□ No

*Q3. Does your registry routinely collect cause of death data?*

*[Single answer]*

□ Yes

□ No

*Q4. After BENCHISTA Phase 1 (period studied 2014-2017), is it feasible for your cancer registry to collect or to continue collecting data on:*

*[one answer per treatment modality]*

□ Chemotherapy Y/N

□ Radiotherapy Y/N

□ Surgery Y/N

□ Immunotherapy Y/N

□ Recurrence Y/N

□ None of the above Y/N

□ Other (open field answer)

*Q5. If you selected YES in any of the above options, please indicate from which year of diagnosis:*

□ Open field answer

*Q6. Does your registry systematically collect or have access to follow-up data for vital status of cancer cases?*

*[Single answer]*

□ Yes, the CR routinely collects complete follow-up data

□ Yes, the CR has access to complete follow-up data but does not routinely collect it

□ Yes, we routinely collect follow-up data, but it is not always complete

□ No, we do not routinely collect follow-up data, but we do have access to it

□ No, we do not routinely collect or have access to follow-up data

□ Other (open field answer)

*Q7. Please indicate how follow-up is achieved by your cancer registry*

*[Multiple choice answer]*

□ Through electronic health records

□ Through hospital or clinical records

□ Linkage with national or regional health databases

□ Linkage to national mortality database or death registries

□ Linkage with administrative databases

□ Linkage to other sources

□ Other sources, please specify (open-field answer)

-- End of survey --

# Table S1. Participating countries in BENCHISTA providing a valid entry to the survey

| **Country** | **n** | **%** |
| --- | --- | --- |
| Belgium | 1 | 1.5 |
| Brazil^a^ | 9 | 13.8 |
| Croatia | 1 | 1.5 |
| Denmark§ | 1 | 1.5 |
| Estonia | 1 | 1.5 |
| France§ | 1 | 1.5 |
| Germany§ | 1 | 1.5 |
| Greece§ | 1 | 1.5 |
| Hungary§ | 1 | 1.5 |
| Ireland | 1 | 1.5 |
| Italy^b^§ | 24 | 36.9 |
| Japan | 1 | 1.5 |
| Netherlands | 1 | 1.5 |
| Norway | 1 | 1.5 |
| Poland^c^ | 2 | 3.1 |
| Portugal§ | 1 | 1.5 |
| Romania§ | 1 | 1.5 |
| Slovakia | 1 | 1.5 |
| Slovenia | 1 | 1.5 |
| Spain^d^§ | 9 | 13.8 |
| Sweden§ | 1 | 1.5 |
| Switzerland§ | 1 | 1.5 |
| United Kingdom^e^ | 3 | 4.6 |
| Total | 65 | 100 |

**Footnote:**

Out of 73 population-based cancer registries (PBCRs) participating in the first phase of the BENCHISTA project, 65 PBCRs from 23 countries provided a complete entry in the survey, which was included in the current data analysis.

^a^ Brazil included answers from regional cancer registry representatives (Aracaju, Barretos, Belem, Campinas, Curitiba, DF, Joao Pessoa, Mato Grosso, and Recife).

^b^ Italy included answers from regional cancer registry representatives (Basilicata, Brianza, Campania, Catania-Messina-Enna, Cremona and Mantua, Emilia Romagna, Friuli Venezia Giulia, Lazio, Marche, Milan, Nuoro, Palermo, Pavia, Piedmont, Puglia, Ragusa and Caltanissetta, Sassari, Siracusa, South Tyrol, Trapani and Agrigento, Trento, Tuscany, Varese, and Veneto).

^c^ Poland included answers from representatives from the Polish National Cancer Registry (PNCR) and the Institute of Mother and Child.

^d^ Spain included answers from regional cancer registry representatives (RETI-SEHOP, Granada, Murcia, Valencia, Basque, Girona, Tarragona, Navarra and Madrid).

^e^ The United Kingdom includes answers from England, Scotland and Wales.

§Population-based childhood cancer specialised registries (n=16): Danish Childhood Cancer Registry. French National Registry of Childhood Solid Tumours – RNTSE. German Childhood Cancer Registry. Nationwide Registry for Childhood Hematological Malignancies – NARECHEM. National Pediatric Cancer Registry of Hungary. Italian PBCRs: Lazio, Marche, Piedmont and Campania. Registo Oncológico Pediátrico Português –ROPP. Romanian National Pediatric Oncology and Hematology Registry. Spanish PBCRs: Comunitat Valenciana, Spanish Registry of Childhood Tumours – RETI-SEHOP, Madrid Childhood Cancer Registry. Swedish Childhood Cancer Registry. Childhood Cancer Registry (ChCR).

# Figure S1. Additional changes reported by PBCRs in assigning tumour stage at diagnosis after participation in BENCHISTA

Better data quality

Pathology and imaging reports now uploaded alongside case records.

Multiple sources used to define staging more precisely.

Better quality of coding.

Coding rules applied for specific tumours (neuroblastoma and rare tumour cases).

Enhanced collaboration

Active use of the BENCHISTA training workshops and related materials.

Clinician involvement and support during staging discussions.

Imaging consultations between regions (Italy) are now routine for paediatric tumours.

Systemic changes

Modifications to databases to allow Toronto stage entry.

Dedicated work files and active search for specific paediatric cancer cases.

Interest in paediatric cancers increased across registries.

Some registries moved to Tier 2 classification.

Increased attention to case verification.

TGs application

CanStaging+ links embeded in reporting forms.

Further implementation of TGs to solid tumours (included in BENCHISTA).

Some registries are planning rollout for additional childhood cancers.

# Table S2. Outline of challenges/limitations reported and potential areas of improvement

|  |  | Areas of improvement | | | | |
| --- | --- | --- | --- | --- | --- | --- |
|  |  | Standardised processes and Electronic Health Record Linkage | Continuous training / educational programme on Toronto Stage | Enhanced interoperability | Governance framework and support to PBCRs | Increased workforce capacity |
| Barriers | Difficulty accessing or retrieving clinical records |  |  |  |  |  |
|  | Incomplete or poor-quality clinical information |  |  |  |  |  |
|  | Required linkage (multi-centre or external) |  |  |  |  |  |
|  | Toronto stage usage / system compatibility |  |  |  |  |  |
|  | Legal / Governance barriers |  |  |  |  |  |

**Footnote:**

PBCRs: Population-based cancer registries.

The topics included in this matrix were reported among respondents when providing an answer to the “Other” category of the question focusing on limitations or challenges encountered when retrieving incidence data and assigning Toronto Stage (Appendix S1 _ Survey question 3).

In terms of legal / governance barriers, and under the BENCHISTA context, specific governance instruments (ethical approvals and a data sharing agreement established since phase 1) were in place to allow international PBCR cooperation.

# Table S3. BENCHISTA Project Working Group

| **Name** | **Institution** |
| --- | --- |
| Peter Baade | Australian Childhood Cancer Registry, Cancer Council Queensland, Brisbane, Australia |
| Leisa O'Neil | Australian Childhood Cancer Registry, Cancer Council Queensland, Brisbane, Australia |
| Florencia Moreno | Argentinian Paediatric Oncology Registry (ROHA) |
| Agustina Chaplin | Argentinian Paediatric Oncology Registry (ROHA) |
| Monika Hackl | Austrian National Cancer Registry, Vienna, Austria |
| Ruth Ladenstein | Children's Cancer Research Institute, Vienna, Austria |
| Elizabeth Van Eycken | Belgian Cancer Registry, Brussels, Belgium |
| Nancy Van Damme | Belgian Cancer Registry, Brussels, Belgium |
| Carlos A. Lima | Registro de Cancer de Base Populacional (RCBP; population-based cancer registry) de Aracaju, Brazil |
| Walmiro Ramos | RCBP de Barretos, Barretos (São Paulo), Brazil |
| Lucrecia Aline Cabral Formigosa | RCBP de Belém, Belém (Pará), Brazil |
| Luciana Ferreira dos Santos | RCBP de Belém, Belém (Pará), Brazil |
| Gil Patrus Mundim Pena | RCBP de Belo Horizonte (Minas Gerais), Brazil |
| Juliana Nativio | RCBP de Campinas, Campinas (São Paulo), Brazil |
| Cyntia Asturian Laporte | RCBP de Curitiba, Curitiba (Paraná), Brazil |
| Cristiana Santos de Menezes Miranda | RCBP de Distrito Federal, Brasília (Federal District), Brazil |
| Cristiane Bastos Daniel | RCBP de Distrito Federal, Brasília (Federal District), Brazil |
| Raimunda Nonata de Paulo | RCBP de Fortaleza (Ceara), Brazil |
| Donaldo B. Veneziano | RCBP de Jahu, Jaú (São Paulo), Brazil |
| Angela Pontes de Aquino | RCBP de Joao Pessoa, João Pessoa (Paraíba), Brazil |
| Paulo Cesar Fernandes de Souza | RCBP de Mato Grosso, Cuiabá (Mato Grosso), Brazil |
| Yana Lopes | Recife Cancer Registry, Recife, Brazil |
| Rejane de Souza Reis | Fundação do Câncer, Rio de Janeiro, Brazil |
| Zdravka Valerianova | Bulgarian Cancer Registry, Sofia, Bulgaria |
| Dobrin Konstantinov | Bulgarian Cancer Registry, Sofia, Bulgaria |
| Sumit Gupta | Children Cancer Registry Pediatric Oncology Group of Ontario (POGO), Toronto, Canada |
| Jason D. Pole | Children Cancer Registry Pediatric Oncology Group of Ontario (POGO), Toronto, Canada |
| Alexandra Airhart | Children Cancer Registry Pediatric Oncology Group of Ontario (POGO), Toronto, Canada |
| Oscar Ramirez | VIGICANCER, Cali, Colombia |
| Elvia Grillo | VIGICANCER, Cali, Colombia |
| Jan Stary | Czech National Cancer Registry, Prague, Czech Republic |
| Jaroslav Sterba | Czech National Cancer Registry, Prague, Czech Republic |
| Lisa L. Hjalgrim | Danish Childhood Cancer Registry and Department of Paediatric Oncology, Copenhagen, Denmark |
| Jeanette Falck Winther | Childhood Cancer Research Group, Danish Cancer Institute & Department of Clinical Medicine, Aarhus University and University Hospital, Aarhus, Denmark |
| Keiu Paapsi | Estonia National Institute for Health Development, Tallinn, Estonia |
| Brigitte Lacour | French National Registry of Childhood Cancer - Solid Tumours, Paris region (INSERM & FRANCIM network), France |
| Emmanuel Desandes | French National Registry of Childhood Cancer - Hematopoietic Malignancies, Paris region (INSERM & FRANCIM network), France |
| Jacqueline Clavel | French National Registry of Childhood Cancer - Hematopoietic Malignancies, Paris region (INSERM & FRANCIM network), France |
| Claire Poulalhon | French National Registry of Childhood Cancer - Hematopoietic Malignancies, Paris region (INSERM & FRANCIM network), France |
| Meike Ressing | German Childhood Cancer Registry, University Medical Center of Johannes Gutenberg-University Mainz, Mainz, Germany |
| Claudia Trübenbach | German Childhood Cancer Registry, University Medical Center of Johannes Gutenberg-University Mainz, Mainz, Germany |
| Eleni T. Petridou | Greek Nationwide Registry for Childhood Hematological Malignancies and Solid Tumours (NARECHEM-ST), Athens, Greece |
| Evdoxia Bouka | Greek Nationwide Registry for Childhood Hematological Malignancies and Solid Tumours (NARECHEM-ST), Athens, Greece |
| Zsuzsanna Jakab | National Childhood Cancer Registry, Budapest, Hungary |
| Miklós Garami | National Childhood Cancer Registry, Budapest, Hungary |
| Rocco Galasso | Basilicata Cancer Registry, Basilicata, Italy |
| Giuseppe Sampietro | Bergamo Cancer Registry, Lombardy, Italy |
| Patrizia Piga | Campania Childhood Cancer Registry, Campania region, Italy |
| Marcella Sessa | Campania Childhood Cancer Registry, Campania region, Italy |
| Milena M. Maule | Childhood Cancer Registry of Piedmont, Piedmont (Turin), Italy |
| Carlotta Sacerdote | Childhood Cancer Registry of Piedmont, Piedmont (Turin), Italy |
| Paola Ballotari | Cremona & Mantova Cancer Registry, Cremona, Mantova (Lombardy), Italy |
| Marilena Ceresola | Cremona & Mantova Cancer Registry, Cremona, Mantova (Lombardy), Italy |
| Ettore Bidoli | Friuli Venezia Giulia Cancer Registry, CRO Aviano National Cancer Institute, Friuli-Venezia Giulia region, Italy |
| Antonina Torrisi | Integrated Cancer Registry CT-ME-EN, Catania-Messina-Enna area (Sicily), Italy |
| Rosalia Ragusa | Integrated Cancer Registry CT-ME-EN, Catania-Messina-Enna area (Sicily), Italy |
| Enrica Santelli | Lazio Region Childhood Cancer Registry |
| Ilaria Cozzi | Lazio Region Childhood Cancer Registry |
| Luca Boni | Liguria Cancer Registry, Ospedale Policlinico San Martino IRCCS, Genova (Liguria), Italy |
| Magda Rognoni | Monza-Brianza Cancer Registry, Monza/Brianza (Lombardy), Italy |
| Rosalba Amodio | Palermo Province Cancer Registry, Palermo (Sicily), Italy |
| Federica Manzoni | Pavia Cancer Registry, Italy |
| Francesco Cuccaro | Puglia Cancer Registry, Puglia region, Italy |
| Danila Bruno | Puglia Cancer Registry, Puglia region, Italy |
| Antonio G. Russo | Registro Tumori ATS della Citta Metropoltiana di Milano, Milan, Italy |
| Federico Gervasi | Registro Tumori ATS della Citta Metropoltiana di Milano, Milan, Italy |
| Maria L. Gambino | Registro Tumori ATS Insubria, Insubria region, Italy |
| Pietro Seghini | Registro Tumori dell’Emilia-Romagna, Unita di Piacenza, Piacenza (Emilia-Romagna), Italy |
| Maria L. Michiara | Registro Tumori dell'Emilia-Romagna, Unita di Parma, Parma (Emilia-Romagna), Italy |
| Luciana Mangone | Registro Tumori dell'Emilia-Romagna, Unita di Reggio Emilia, Reggio Emilia (Emilia-Romagna), Italy |
| Claudia Cirilli | Registro Tumori dell'Emilia-Romagna, Unita di Modena, Modena (Emilia-Romagna), Italy |
| Stefano Ferretti | Registro Tumori dell'Emilia-Romagna, Unita di Ferrara, Ferrara (Emilia-Romagna), Italy |
| Federica Zamagni | Registro Tumori dell'Emilia-Romagna, Unita della Romagna, IRCCS IRST Meldola, Romagna area (Meldola), Italy |
| Fabio Falcini | Registro Tumori dell'Emilia-Romagna, Unita della Romagna, IRCCS IRST Meldola, Romagna area (Meldola), Italy |
| Eugenia Spata | Registro Tumori di Ragusa e Caltanissetta, Ragusa and Caltanissetta (Sicily), Italy |
| Sonia Manasse | Registro Tumori Regione Marche, Marche region, Italy |
| Paolo Coccia | Registro Tumori Regione Marche, Marche region, Italy |
| Fabrizio Stracci | Registro Tumori Umbria, Umbria region, Italy |
| Daniela Piras | Registro Tumori Sassari, Sassari (Sardinia), Italy |
| Pasquala Pinna | Registro Tumori Nuoro, Nuoro (Sardinia), Italy |
| Francesca Bella | Siracusa Cancer Registry, Siracusa (Sicily), Italy |
| Andreas Klaus Bulatko | South Tyrol Cancer Registry, Italy |
| Adele Caldarella | Toscana Cancer Registry, Florence (Tuscany), Italy |
| Teresa Intrieri | Toscana Cancer Registry, Florence (Tuscany), Italy |
| Tiziana Scuderi | Registro Tumori Trapani Agrigento, Trapani and Agrigento (Sicily), Italy |
| William Mantovani | Trento Cancer Registry, Trento, Italy |
| Maria Adalgisa Gentilini | Trento Cancer Registry, Trento, Italy |
| Manuel Zorzi | Veneto Cancer Registry, Veneto region (Padova/Venezia), Italy |
| Stefano Guzzinati | Veneto Cancer Registry, Veneto region (Padova/Venezia), Italy |
| Tracey Brennan | National Cancer Registry Ireland, Cork, Ireland |
| Tomohiro Matsuda | National Cancer Centre, Tokyo, Japan |
| Kayo Nakata | Osaka Cancer Registry, Osaka, Japan |
| Kyu-Won Jung | National Cancer Center Korea |
| Marika Gurupta | Latvian Cancer Registry |
| Jelena Rascon | Lithuanian Cancer Registry |
| Miriam J. Azzopardi | Malta National Cancer Registry, Pietà, Malta |
| Tom Borge Johannesen | Norwegian Cancer Registry, Oslo, Norway |
| Aina H. Dahlen | Norwegian Cancer Registry, Oslo, Norway |
| Einar Stensvold | Norwegian Cancer Registry, Oslo, Norway |
| Joanna Didkowska | Polish National Cancer Registry |
| Ursula Wojciechowska | Polish National Cancer Registry |
| Anna Raciborska | Polish Childhood Cancer Registry, Institute of Mother and Child, Warsaw, Poland |
| Ana M. Ferreira | Portuguese Pediatric Cancer Registry, Lisbon, Portugal |
| Gabriela Caldas | Portuguese Pediatric Cancer Registry, Lisbon, Portugal |
| Maria José Bento | RON – Registro Oncologico Nacional |
| Mihaela Bucurenci | Romanian Child Cancer Registry, Balotești, Ilfov County, Romania |
| Daniela Coza | Romanian Child Cancer Registry, Balotești, Ilfov County, Romania |
| Miroslava Makohusova | National Institute of Children’s Diseases, Bratislava, Slovakia |
| Gabriela Valachova | National Health Information Center, Slovakia |
| Vesna Zadnik | Cancer Registry of Republic of Slovenia, Ljubljana, Slovenia |
| Tina Žagar | Institute of Oncology Ljubljana |
| Arantza Lopez de Munain | Basque Country, Euskadi-CIBERESP Cancer Registry, Vitoria Gasteiz, Basque Country, Spain |
| Fernando Almela-Vich | Childhood and Adolescents Cancer Registry - CISCV, Valencia region, Spain |
| Noura Jeghalef-El Karoui | Childhood and Adolescents Cancer Registry - CISCV, Valencia region, Spain |
| Aina Romaguera Palomé | Girona Cancer Registry, Girona, Catalonia, Spain |
| Rafael Marcos Gragera | Girona Cancer Registry, Girona, Catalonia, Spain |
| Maria José Sanchez | Granada Cancer Registry, Granada, Andalucia, Spain |
| Nuria Aragones | Madrid Childhood Cancer Registry, Madrid, Spain |
| David Parra-Blazquez | Madrid Childhood Cancer Registry, Madrid, Spain |
| Maria Dolores Chirlaque | Murcia Cancer Registry, Murcia, Spain |
| Ricardo J. Vaamonde | Murcia Cancer Registry, Murcia, Spain |
| Marcela Guevara | Navarra Cancer Registry, Pamplona (Navarra), Spain |
| Elena Pardo | Spanish Registry of Childhood Tumours (RETI-SEHOP), University of Valencia, Spain |
| Rafael Peris-Bonet | Spanish Registry of Childhood Tumours (RETI-SEHOP), University of Valencia, Spain |
| Adela Cañete Nieto | Paediatric Oncology and Hematology Unit, Hospital UiP La Fe, Valencia, Spain |
| Marià Carulla | Tarragona Cancer Registry, Tarragona, Catalonia, Spain |
| Päivi Lähteenmäki | Swedish Childhood Cancer Registry – SCCR, Karolinska Institute, Stockholm, Sweden |
| Claudia E. Kuehni | Childhood Cancer Registry (ChCR), Institute of Social and Preventive Medicine, University of Bern, Bern, Switzerland |
| Sophia Della Valle | Childhood Cancer Registry (ChCR), Institute of Social and Preventive Medicine, University of Bern, Bern, Switzerland |
| Shelagh M. Redmond | Childhood Cancer Registry (ChCR), Institute of Social and Preventive Medicine, University of Bern, Bern, Switzerland |
| Otto Visser | The Netherlands Cancer Registry, Utrecht, The Netherlands |
| Henrike Karim-Kos | Princess Máxima Center for Pediatric Oncology, Utrecht, The Netherlands |
| Sarah Stevens | National Disease Registration Service, Transformation Directorate, NHS England, London, UK. |
| Lucy Irvine | National Disease Registration Service, Transformation Directorate, NHS England, London, UK. |
| Charles A. Stiller | National Disease Registration Service, Transformation Directorate, NHS England, London, UK. |
| Anna Gavin | Centre for Public Health, Belfast, Northern Ireland |
| Deidre Fitzpatrick | Northern Ireland Cancer Registry, Queen’s University, Belfast, Northern Ireland |
| Damien Bennett | Northern Ireland Cancer Registry, Queen’s University, Belfast, Northern Ireland |
| David S. Morrison | Scottish Cancer Registry, Edinburgh, Scotland |
| Karen Smith | Scottish Cancer Registry, Edinburgh, Scotland |
| Claire Manson | Scottish Cancer Registry, Edinburgh, Scotland |
| Dyfed Wyn Huws | Welsh Cancer Intelligence and Surveillance Unit, Public Health Wales, Cardiff, Wales |
| Stephanie Smits | Welsh Cancer Intelligence and Surveillance Unit, Public Health Wales, Cardiff, Wales |
| Dawn Allan | Welsh Cancer Intelligence and Surveillance Unit, Public Health Wales, Cardiff, Wales |

**Other Representatives**

| **Name** | **Institution** |
| --- | --- |
| Angela Polanco | NIHR (National Institute for Health and Care Research), London, UK |
| Riccardo Capocaccia | Epidemiologia & Prevenzione, Rome, Italy |
| Andrea Di Cataldo | Universita degli Studi di Catania, Catania (Sicily), Italy |
| Meric Klein | Belgian Cancer Registry, Brussels, Belgium |
| Maciej Trojanowski | Greater Poland Cancer Registry, Department of Cancer Prevention and Epidemiology, Poland |

Footnote: Historical record of BENCHISTA Project Working Group Members and other representatives.
